# Renal Dysfunction in Patients with Left Ventricular Assist Device

**DOI:** 10.14797/mdcvj.1146

**Published:** 2022-09-06

**Authors:** Lamees I. El Nihum, Nina Manian, Priya Arunachalam, Qasim Al Abri, Ashrith Guha

**Affiliations:** 1 Methodist DeBakey Heart & Vascular Center, Houston Methodist, Houston, Texas, US; 2 Texas A&M College of Medicine, Bryan, Texas, US

**Keywords:** LVAD, left ventricular assist device, renal dysfunction

## Abstract

Late-stage heart failure and renal dysfunction are often seen in conjunction. Cardiorenal syndrome (CRS) describes the complex interaction between the two disease states. Early literature described the pathophysiology of CRS as related only to reduced cardiac output and decreased renal perfusion. Recent literature suggests a more multifaceted mechanism. Left ventricular assist devices (LVAD), used as bridge-to-transplant and destination therapy in patients with heart failure, impact not only cardiac function but also renal function, especially in those patients with preoperative renal dysfunction. The mechanism by which LVAD implantation affects renal function is complex and understated in early literature. In this review, we discuss the pathogenesis of CRS, the impact of preoperative renal dysfunction in patients undergoing LVAD implantation, and the effect of LVAD implantation on postoperative renal function.

## Introduction

The prevalence of heart failure (HF) in the United States (US) continues to rise. An estimated 6.2 million Americans over 20 years of age had HF between 2013 and 2016 compared to approximately 5.7 million between 2009 and 2012.^1^ Mechanical circulatory support is increasingly used to provide bridge-to-transplant and destination therapy in patients with advanced HF.^2^

Renal dysfunction of varying degrees is a common comorbidity in HF patients and may be caused by intrinsic or preexisting renal disease, cardiorenal syndrome (CRS), or a combination of the two. The pathophysiology of CRS in HF has been widely studied and refers to the complex interaction between HF and ensuing renal dysfunction caused by reduction in cardiac output and renal perfusion. In this review, we discuss the pathogenesis of CRS, examine the impact of renal dysfunction on outcomes after left ventricular assist device (LVAD) therapy, and assess the effect of LVAD on preoperative renal dysfunction.

## Pathophysiology of Cardiorenal Syndrome

In CRS, acute or chronic dysfunction of the heart can lead to acute or chronic dysfunction in the kidney and vice versa.^3^ Advanced heart failure is characterized by increased pressures in the right and left atria with decreased cardiac output leading to increased glomerular pressure, which in turn leads to decreased filtration rate.^4,5,6^ Recent research shows CRS is a broad term describing an aberrancy in either renal or cardiac function that leads to dysfunction in the other organ. Neurohormonal activation, low systemic blood pressure, and increase intraperitoneal pressures also contribute to the pathophysiology of CRS.^7^

Decreased cardiac output in HF has been widely considered the basis for renal damage in CRS. However, the latest research suggests decreased cardiac output has a very small role in the pathophysiology of renal dysfunction.^8^ Kidneys have sophisticated mechanisms of autoregulation that stabilize renal function in the setting of reduced cardiac output. Evidence shows that other factors, such as low systemic blood pressure, venous congestion, increased intrabdominal pressures, and maladaptive neurohormonal activation, more significantly contribute to renal dysfunction in patients with HF. In fact, some studies suggest low systemic blood pressure as the factor most strongly associated with renal dysfunction in patients with HF.^9^

CRS type 1, also known as acute CRS, occurs when an acute worsening of cardiac function, such as acute decompensated HF, leads to acute kidney injury.^7^ The acute decrease in cardiac output leads to decreased renal plasma flow and perfusion. The initial response of the kidneys to this decreased perfusion pressure is relative preservation of glomerular filtration rate (GFR) due to the increase in efferent arteriolar resistance by angiotensin II.^7^ Subsequently, a neurohormonal response is triggered that leads to activation of the sympathetic nervous system and the renin-aldosterone-angiotensin system (RAAS).^7^ As injury continues, the neurohormonal pathways become inappropriately activated and maladaptive, resulting in increased salt and water retention in the tubules and systemic vasoconstriction.^7^ Angiotensin II, the product of the RAAS pathway, promotes the formation of reactive oxygen species via activation of nicotinamide adenine dinucleotide phosphate oxidase, resulting in microvascular damage and furthering cardiomyocyte loss and worsening HF.^7^ These maladaptive changes perpetuate the cycle of damage in CRS.^7^ In CRS type 2, which is a chronic form of CRS, chronic abnormalities in cardiac function, including decreased cardiac output and increased central venous pressure, result in increased renal vein pressure, decreasing GFR.^7^

The Evaluation Study of Congestive Heart Failure and Pulmonary Artery Catheterization Effectiveness (ESCAPE) demonstrated that right atrial pressure was associated with renal impairment, and increased central venous pressure was associated with reduced GFR.^6,10^ Additionally, activation of the sympathetic nervous system and RAAS activation exacerbate both cardiac and kidney dysfunction.

### Renal Dysfunction and Cardiac Surgery

**Figure 1:**
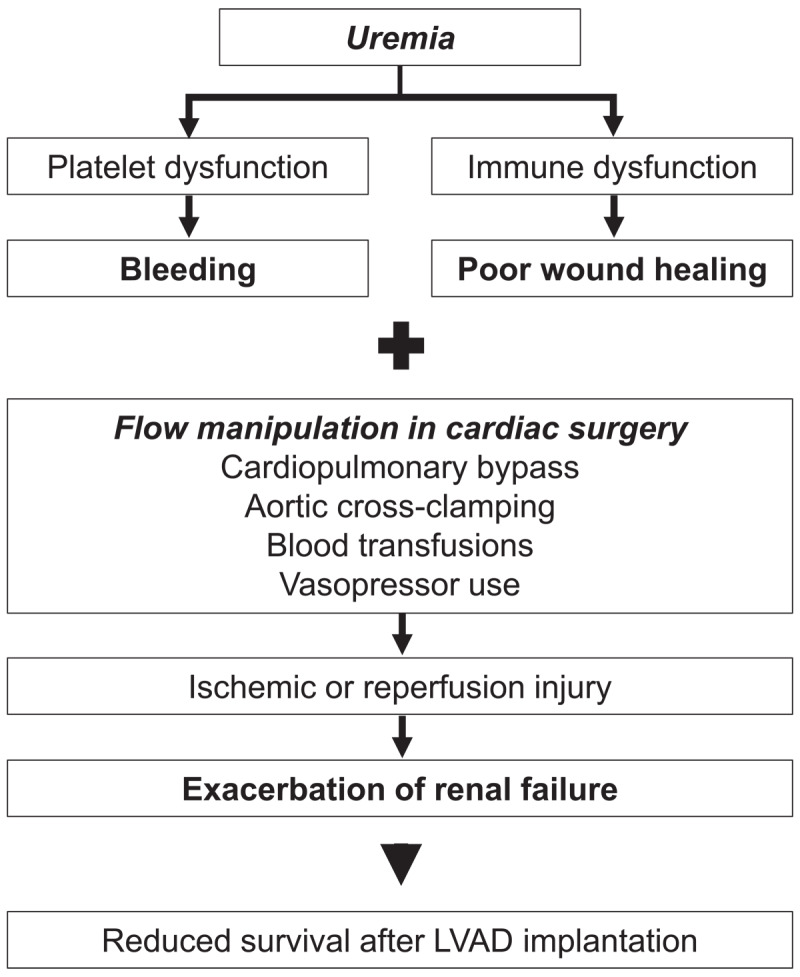
Preoperative renal dysfunction and cardiac surgery. LVAD: left ventricular assist device

Presence of preoperative renal dysfunction and the associated metabolic derangements can lead to complications during cardiac surgery, including in patients undergoing LVAD implantation (Figure 1). Renal dysfunction is associated with uremia, acidosis, and anemia. Platelet dysfunction as a result of uremia leads to increased bleeding. Additionally, uremia leads to immune dysfunction, thereby affecting the transposition of cells necessary for wound healing after cardiac surgery and leading to poor wound healing.^11^

Renal dysfunction may be further exacerbated during cardiac surgery. Cardiac surgeries manipulate flow intraoperatively through cardiopulmonary bypass, aortic cross-clamping, blood transfusions, and vasopressor use, all of which increase the risk of postoperative kidney dysfunction. The manipulation of flow during these steps of cardiac surgery alters renal perfusion and may lead to ischemic or reperfusion injury, exacerbating renal failure.^12^

### Impact of Renal Dysfunction on Immediate LVAD Outcomes

Many LVAD recipients have concurrent renal dysfunction as a consequence of CRS Stage 3 chronic kidney disease (CKD) (GFR < 60 mL/min/1.73m^2^) has been reported in up to one-fourth of patients with chronic heart failure and in patients undergoing LVAD implantation.^13^

Preoperative renal dysfunction is associated with progressively reduced survival after LVAD implantation. A systematic review of seven studies and 26,652 patients who underwent LVAD implantation demonstrated a significant increase in all-cause mortality in patients with impaired renal function preoperatively.^14^ In addition, an analysis of the INTERMACS database between 2006–2014 investigated 15,754 patients who received mechanical circulatory support, and 12.3% developed kidney dysfunction needing dialysis.^15^ Another study conducted a 180-day end-point assessment of 332 patients in bridge-to-transplant and continued access protocols.^16^ This study revealed that 9.6% of LVAD recipients in the combined bridge-to-transplant and continued-access protocol developed kidney dysfunction.^17^ In addition, Asleh et al. examined use of renal replacement therapy (RRT) in a study of 354 patients who underwent LVAD implantation between 2007–2017.^18^ Of these patients, 15% required in-hospital RRT following LVAD implantation.^18^ Characteristics of these patients included higher preoperative Charlson Comorbidity Index values, higher Model for End-Stage Liver Disease scores, higher right atrial pressure, higher estimated 24-hour urine protein levels, and lower preoperative estimated GFR and measured GFR using _125_I-iothalamate clearance when compared to those who did not require RRT.^18^ Of the patients requiring in-hospital RRT, 33% had renal recovery, 33% required outpatient hemodialysis, and 33% died before hospital discharge.^18^ The study further concluded that higher preoperative creatinine levels, higher 24-hour urine protein levels, and higher mean right atrial pressure and longer cardiopulmonary bypass time were independent predictors of RRT after LVAD.^18^

### LVAD and Renal Recovery

**Figure 2:**
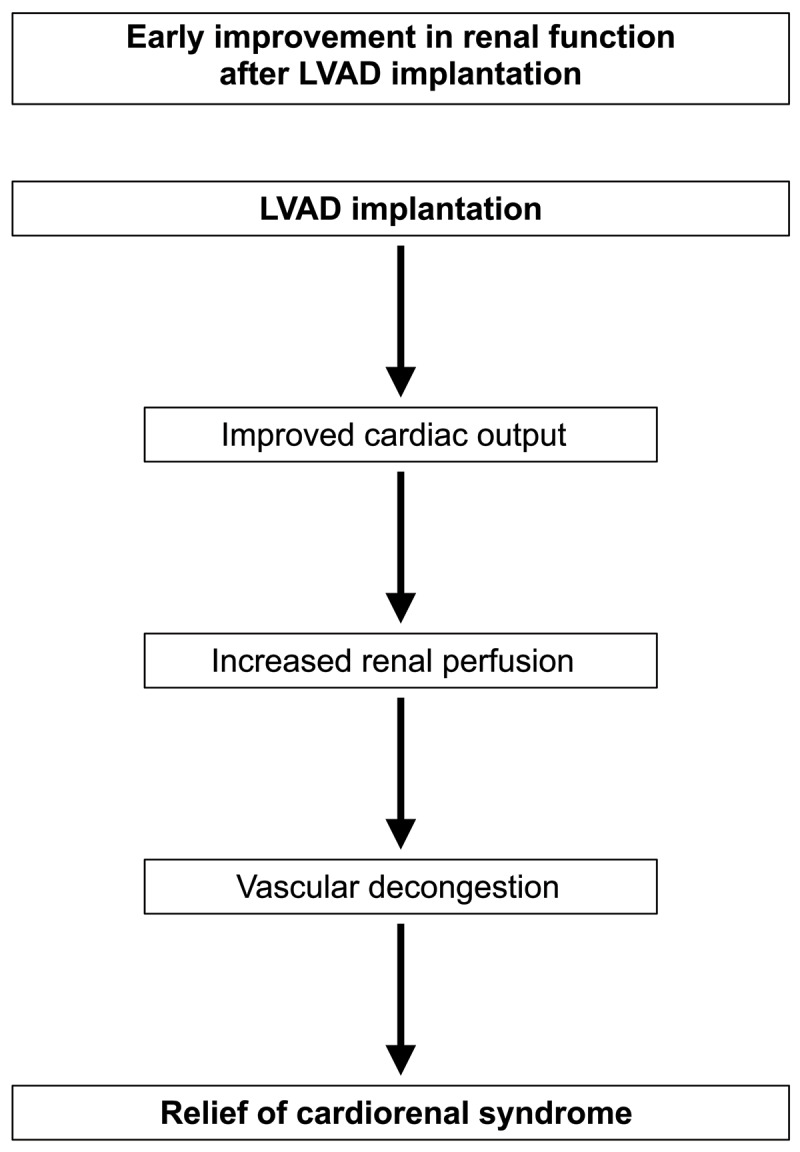
Relief of cardiorenal syndrome. LVAD: left ventricular assist device

Previously, it was thought that renal dysfunction in HF patients was primarily due to decreased cardiac output. Under this school of thought, LVAD implantation should improve CRS due to improved cardiac output and renal perfusion (Figure 2). However, recent studies have noted the minimal effect of cardiac output on renal function in patients with heart failure and the more prominent effect of factors such as low systemic blood pressure and central venous congestion.^8^ Despite the latest research, studies show 50% to 60% of patients experience some form of early kidney recovery following LVAD placement.^19^ In one study involving 238 LVAD recipients with reduced preoperative GFR, 43% of bridge-to-transplant and 57% of destination therapy patients recovered renal function to a GFR > 60 mL/min/1.73m^2^ within the first year after implantation.^20^ Improvement occurred most significantly within the first 3 months following LVAD implantation, after which mean GFR declined slightly yet remained significantly higher than baseline at 1 year post-implantation.^20^ However, while some patients experience this improvement in kidney function, many remain in a state of kidney dysfunction or only experience short-lived improvement in kidney function despite LVAD implantation.^13^

Notably, long-term outcomes tend to culminate in a decline in renal function after the first year.^13^ One study revealed that while 48.9% of patients initially experienced improved kidney function, they experienced a decline in GFR after one year.^21^ Interestingly, patients without kidney dysfunction prior to LVAD implantation had a decline in kidney function at 1 year, while patients with kidney dysfunction prior to LVAD implantation maintained a GFR above the baseline.^21^ Another study compared kidney function in LVAD patients with pre-LVAD GFR < 40 mL/min/1.73m^2^ versus > 40 mL/min/1.73m^2^.^22^ In both groups GFR increased significantly at 1 month and then declined; at 1 year, patients with pre-LVAD GFR < 40 maintained a higher-than-baseline GFR while those with pre-LVAD GFR >40 returned to pre-LVAD baseline.^22^ These studies may indicate that patients are unable to maintain improvement in kidney function after LVAD implantation.

**Figure 3:**
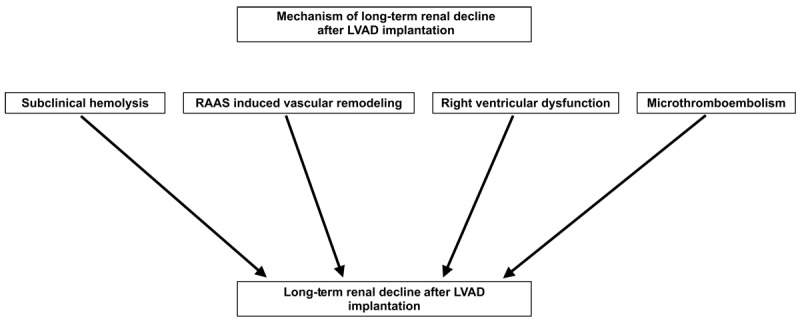
Mechanisms of long-term renal decline after LVAD implantation. LVAD: left ventricular assist device; RAAS: renin-aldosterone-angiotensin system

The mechanism of long-term renal damage post-LVAD implantation is multifactorial (Figure 3). Continuous flow LVADs are preferable to the older pulsatile flow LVADs due to increased durability and more practical dimensions.^23^ However, continuous flow LVADs are less physiologic and lead to neurohormonal alterations that affect the kidneys. Studies show that the lack of pulsatile flow leads to decreased sensitivity of arterial baroreceptors to fluctuations in flow.^24^ Arterial baroreceptors physiologically respond to changes in flow and inhibit the sympathetic nervous system.^24^ However, with continuous flow, the arterial baroreceptors are unable to respond, leading to increased stimulation of the sympathetic nervous system and subsequent activation of the RAAS pathway.^24^ A study by Ootaki et al. showed that increased activation of the RAAS pathway in calf models with continuous LVADs led to detrimental histologic changes in the kidney.^25^ These changes included wall thickening of the calves’ arcuate and interlobular arteries secondary to RAAS activation, indicating severe renal periarteritis.^25^ Additionally, LVADs can cause subclinical hemolysis, and the breakdown products, hemoglobin and iron, in excess are nephrotoxic and can lead to pigment nephropathy. One study found an association between the severity of hemolysis, measured by lactate dehydrogenase levels, and the occurrence of acute kidney injury and CKD.^26^

Another mechanism for long-term renal decline after LVAD implantation is right ventricular dysfunction leading to decreased renal perfusion. Right ventricular preload is dramatically increased in patients with LVADs due to increased cardiac index from improved left ventricular function.^23^ The increased venous return to the heart leads to right ventricular myocardial stress and can lead to right ventricular dilation with subsequent tricuspid regurgitation, further enhancing the right ventricular weakening.^23^ The right ventricular dysfunction causes increased central venous pressure, increased renal venous pressure, and decreased GFR.^23^

Jawaid et al. note that early improvement in renal function within 6 months of LVAD placement is noted in up to 60% of patients and is attributed to relief of CRS and decongestion as well as reductions in vascular resistance within the kidney due to changes in renal venous pressure, RAAS, and sympathetic activity.^19^ Recent literature suggests the early improvement in renal function seen in many studies may not be true improvement in renal function. In many of the studies outlined, estimated GFR (eGFR) is measured by serum creatinine. Serum creatinine is released by muscle cells. Many patients who receive LVAD devices are critically ill and have significant muscle wasting. In these patients with decreased muscle mass, serum creatinine over-estimates eGFR. One review paper suggested patients experience additional muscle wasting immediately following LVAD implantation as they recover from surgery and are bed-bound.^27^ This muscle wasting, and therefore decreased serum creatinine as a result of lost muscle mass, may explain the acute improvement in eGFR seen in many studies.

A combined prospective and retrospective cohort study found that serum cystatin C may be a more accurate marker of renal function in LVAD patients.^28^ Cystatin C is released by all nucleated cells, not just muscle cells, and is therefore less impacted by the sarcopenia seen in LVAD patients. The study found serum cystatin C levels are more strongly associated with the primary risk end point, which is right ventricular failure and need for renal replacement therapy, than serum creatinine levels. While serum cystatin C may seem to be the answer, one recent study found that serum cystatin C levels are in fact associated with muscle mass in patients with HF.^29^ This suggests the need for alternative renal biomarkers to evaluate renal function in LVAD patients.

Chronically, within 1 year of LVAD placement, renal deterioration is noted in 79% to 84% of patients and may be attributed to RAAS-induced adverse vascular remodeling, subacute hemolysis with glomerular pigment deposition, and progressive right ventricular failure.^19^ Late dysfunction of greater than 1 year after LVAD placement is noted in up to 50% of patients and may be attributed to RAAS-induced adverse vascular remodeling, subacute hemolysis with glomerular pigment deposition, progressive right ventricular failure, and microthromboembolism.^19^

## End Stage Renal Disease And LVAD

End stage renal disease (ESRD) is defined as having received maintenance dialysis or kidney transplant for treatment of CKD.^14^ Both patients with CKD and patients with ESRD have been found to have a higher 1-year mortality after LVAD implantation compared to patients with normal renal function.^30^ Of note, patients with CKD who require RRT during their LVAD implantation hospitalization have a higher 1-year mortality than patients with CKD who do not require RRT. Patients with ESRD prior to LVAD implantation have been demonstrated to have extremely poor prognoses.^31^ These patients have a higher 1-year mortality rate compared to patients with CKD.^30^ An 11-year study following LVAD recipients with ESRD revealed 81.9% mortality among patients with ESRD versus 36.4% among patients without ESRD.^31^ Median time to death among ESRD LVAD recipients was 16 days after implantation versus 2,125 days after implantation among those without ESRD.^31^ The precise mechanism by which renal failure affects LVAD mortality outcomes is unknown. However, it is likely related to the build-up of toxic metabolites, such as urea, uremic toxins, and proteins (ie, fibroblast growth factor 23), that are associated with an increased risk of cardiovascular events.

Patients with LVAD requiring hemodialysis in the acute or long-term setting pose a unique challenge in terms of maintaining hemodynamic monitoring, normotension, right ventricular function, and other hemodynamic and physiological factors during dialysis in order to minimize morbidity and mortality.^32^ Due to the challenges of identifying local dialysis centers willing to accept LVAD patients, peritoneal dialysis has been suggested as an option for LVAD patients requiring long-term outpatient RRT.^18^

## Conclusion

CRS is a complex interaction of HF and kidney dysfunction that both affects and is affected by LVAD implantation. In the short term, LVAD implantation may serve to ameliorate renal dysfunction in patients with advanced HF. However, deterioration in kidney function is often seen in long-term LVAD patients, though mechanisms for its development are poorly understood. Carefully selected patients with advanced HF and renal dysfunction have successful outcomes after LVAD implantation, thus renal dysfunction in itself should not be considered an absolute contraindication.

## Key Points

- The pathophysiology of cardiorenal syndrome (CRS) involves pathologic neurohormonal activation, decreased systemic blood pressure, and increased intraperitoneal pressures.
- Preoperative renal dysfunction leads to complications during left ventricular assist device (LVAD) implantation surgery because of renal dysfunction-associated uremia, anemia, and acidosis.
- Based on early school of thought, LVAD implantation should improve renal function because of increased cardiac output. In contrast, recent literature shows LVAD implantation contributes to long-term decline in renal function.
- Some studies show renal dysfunction improves in the first 6 months after LVAD implantation. Recent literature suggests the early renal function improvement may be overstated, as post-LVAD patients have muscle wasting and creatinine clearance is not an accurate estimation of glomerular filtration rate in these patients.
- Patients with end stage renal disease (ESRD) have significantly worse outcomes post-LVAD implantation compared to patients with normal renal function and even compared to patients with chronic kidney disease. The mechanism by which ESRD worsens outcomes is unknown but is likely related to the accumulation of toxic metabolites, which increase the risk of adverse cardiovascular events.
